# Study on the Relationship between Particulate Methane Monooxygenase and Methanobactin on Gold-Nanoparticles-Modified Electrodes

**DOI:** 10.3390/molecules29061270

**Published:** 2024-03-13

**Authors:** Boxin Dou, Mingyu Li, Lirui Sun, Jiaying Xin, Chungu Xia

**Affiliations:** 1 College of Food Engineering, Key Laboratory of Food Science and Engineering of Heilongjiang Ordinary Higher Colleges, Harbin University of Commerce, Harbin 150000, China; mingxiaoyua@163.com (M.L.); jiangxing0423@163.com (L.S.); xinjiayingvip@163.com (J.X.); 2 State Key Laboratory for Oxo Synthesis and Selective Oxidation, Lanzhou Institute of Chemical Physics, Chinese Academy of Sciences, Lanzhou 730000, China; 15663705260@163.com

**Keywords:** particulate methane monooxygenase, methanobactin, direct electrochemistry, gold nanoparticles, electrochemical

## Abstract

(1) Background: Particulate methane monooxygenase (pMMO) has a strong dependence on the natural electron transfer path and is prone to denaturation, which results in its redox activity centers being unable to transfer electrons with bare electrodes directly and making it challenging to observe an electrochemical response; (2) Methods: Using methanobactin (Mb) as the electron transporter between gold electrodes and pMMO, a bionic interface with high biocompatibility and stability was created. The Mb-AuNPs-modified functionalized gold net electrode as a working electrode, the kinetic behaviors of pMMO bioelectrocatalysis, and the effect of Mb on pMMO were analyzed. The CV tests were performed at different scanning rates to obtain electrochemical kinetics parameters. (3) Results: The values of the electron transfer coefficient (α) and electron transfer rate constant (*k_s_*) are relatively large in test environments containing only CH_4_ or O_2_. In contrast, in the test environment containing both CH_4_ and O_2_, the bioelectrocatalysis of pMMO is a two-electron transfer process with a relatively small α and *k_s_*; (4) Conclusions: It was inferred that Mb formed the complex with pMMO. More importantly, Mb not only played a role in electron transfer but also in stabilizing the enzyme structure of pMMO and maintaining a specific redox state. Furthermore, the continuous catalytic oxidation of natural substrate methane was realized.

## 1. Introduction

Methane monooxygenases (MMO) are produced by methanotrophs who can use methane as a substrate for growth which has two forms of existence. One is the soluble methane monooxygenase (sMMO), a cytoplasmic MMO investigated extensively by several research groups. The other is the particulate methane monooxygenase (pMMO), a membrane-bound MMO and a key and rate-limiting enzyme.

However, pMMO is much less well characterized, mainly due to its unusual activity instability, which is known to be very difficult to study. The pMMO-catalyzed CH_4_ oxidation process is accompanied by electron transfer, and electrochemical methods can solve the problem of extracellular coenzyme regeneration. However, pMMO is susceptible to denaturation and causes electrode passivation on the surface of conventional electrodes [1,2,3]. At the same time, pMMO has a strong dependence on the natural electron transfer pathway, making it impossible for the redox-active center to transfer electrons directly with the electrode. Therefore, it is difficult to observe the electrochemical response of pMMO on conventional bare gold electrodes. While the small peptide called methanobactin (Mb) secreted by methanotrophs can capture Cu(II), it can also combine with pMMO to form pMMO complexes in the intima and participate in electron transfer of process catalyzed by pMMO. In addition, the Mb-Cu complex has peroxidase (POD) and superoxide dismutase (SOD) activity, which makes Mb or Mb-Cu complex be used as the superoxide anion scavenger to protect pMMO and maintain a particular redox state in the presence of oxygen. Mb contains sulfhydryl groups (or disulfide), which can adsorb the stability of elemental gold.

Nanogold, also known as Au nanoparticles (AuNPs), refers to gold particles with a diameter of 1~100 nm, which can exist in a stable form in solution and is one of the most commonly used types of nanometallic materials. The AuNPs particles, as commonly used solid enzyme carriers, have many advantages, such as a high specific surface area, excellent conductivity, controllable particle size, and accessible surface modification [4,5]. However, when AuNPs are in direct contact with enzymes or other proteinaceous small molecules, the interaction between AuNPs particles and the peptide ligands near the active center of the enzyme may result in a distortion of the enzyme or other proteinaceous small molecule’s active center conformation, leading to a decrease in catalytic activity or even inactivation. Attempts were made to adjust the hydrophobic environment around AuNPs by sulfhydryl reagents to obtain Mb-functionalized AuNPs that are conducive to electron transfer and direct electrochemical studies of pMMO. Thus, the present experiments considered a hybrid modification approach to solve these problems. Most important, when assembled on the surface of AuNPs, long-chain sulfhydryl compounds may be bent and entangled. Therefore, the short-chain bifunctional reagents mixed with Mb were selected to modify the surface binding sites of AuNPs in this experiment.

Mb is indispensable if direct electrochemical studies of pMMO are to be achieved. pMMO has a strong dependence on the natural electron transfer pathway. Moreover, it is prone to denaturation on the surface of conventional electrodes. It causes electrode passivation, resulting in the inability of its redox-active center to transfer electrons directly with the electrodes, which makes it challenging to observe the electrochemical response of pMMO at conventional bare gold electrodes. Mb can be combined with pMMO to form a pMMO complex, which participates in the electron transfer in the pMMO-catalyzed CH_4_ oxidation process.

Moreover, Mb can increase the amount of electrons flowing to pMMO and may play a role in transferring the electrons from the electron donor to pMMO in pMMO-catalyzed CH_4_ oxidation. In a superoxide environment (in the presence of no substrate), Mb and Mb-Cu possess SOD and POD activities, which can stabilize the activity of the pMMO enzyme so that the pMMO can be maintained in a particular redox state. Based on the above functional properties of Mb, it provides a new idea to study the enzymatic properties of pMMO and to achieve the continuous catalysis of substrate oxidation by pMMO. However, if the biological microenvironment is lost outside the cell and the coenzymes NADH^+^H^+^ are constantly consumed as electron donors, catalytic oxidation cannot be continued [6].

In this experiment, an Mb-functionalized AuNPs layer-by-layer self-assembled modified electrode was utilized as a biological bridge for pMMO to undergo electron transfer on the electrode surface. The Mb on the surface of electrodes also protected the natural conformation and biological activity of pMMO. It enhanced the reversibility of redox reactions on the electrode surfaces. It improved the load capacity of pMMO, and the interface of enzyme activity centers in contact with the electrode through the high specific areas of the AuNPs. The direct electrochemistry of pMMO was determined in different contents of oxygen species to explore how Mb interacted with pMMO, and the relevant electrochemical kinetic parameters were obtained for the direct electrocatalytic redox reaction of pMMO.

## 2. Results and Discussion

### 2.1. Impact Tests of Different CV Scanning Speed

**Figure 1:**
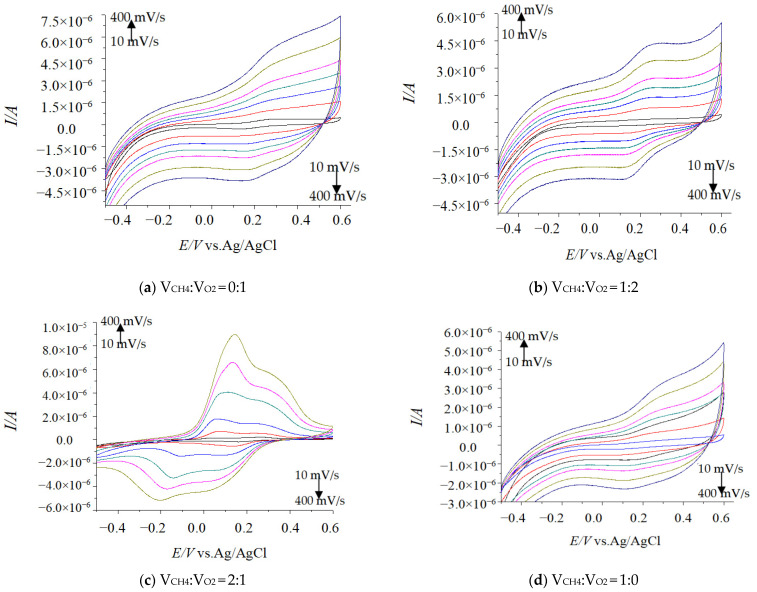
Effect CV results with different scanning speeds (vs. Ag/AgCl).

**Figure 2:**
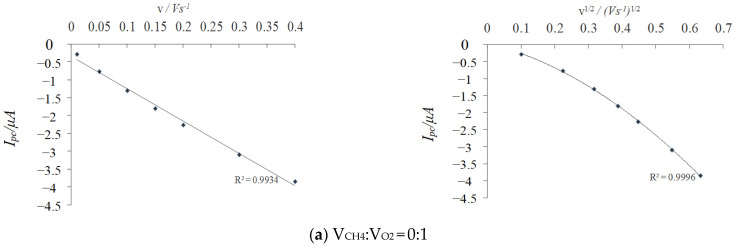

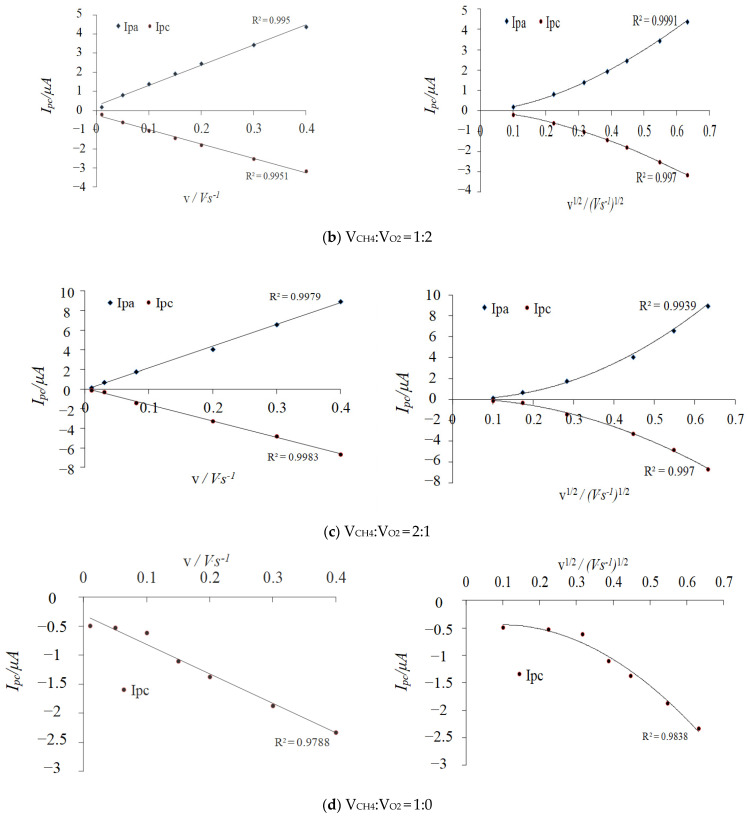
Relationship between peak current (*Ip*) and scanning speed (*v* and *v*^1/2^).

Under different environments, the CV experiments were tested at different scanning rates and different CV peak shapes appeared in each test (Figure 1). In the potential window −0.2 V to + 0.6 V (vs. Ag/AgCl), the peak current (*Ip*) showed a linear correlation with the scanning rates (*v* and *v*^1/2^) at less than 400 mV·s^−1^ (Figure 2), which were consistent with the surface kinetics model of electrochemical reaction [7].

### 2.2. Effect of Dissolved CH_4_ and O_2_ Volumes Ratio on Electrochemical Kinetics Parameters

#### 2.2.1. Transferred Electron Number (*n*)

The Laviron equation is suitable for non-diffusion control reactions. The transferred electron number (*n*) can be calculated based on the integral area (*Q*) and scanning rates (*v*). In the formula, (mol/cm^2^) is the apparent coverage of the surface modifier of the working electrode; *A* (cm^2^) is the surface area of the working electrode; *R* is the gas constant, 8.314 J/(mol·K); *F* is the Faraday constant, and its value is 96,500 C·mol^−1^; *T* indicates the thermodynamic temperature and its value is 298.15 (25 °C). The effect of the dissolved CH_4_ and O_2_ volume ratio on the electrochemical kinetics parameters is shown in Table 1.

(1) $$Ip=\;\frac{n^{2}F^{2}AFv}{4RT}=\frac{nFQv}{4RT}$$

#### 2.2.2. Electron Transfer Coefficient (*α*)

**Figure 3:**
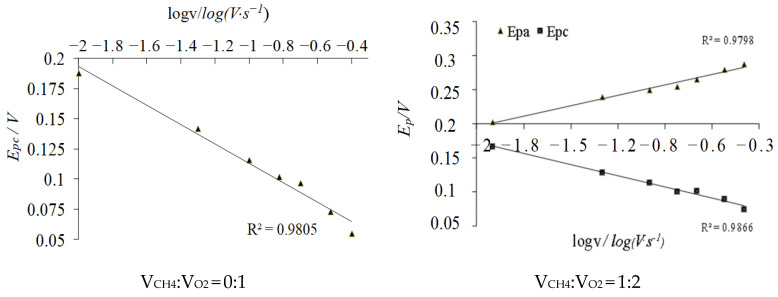

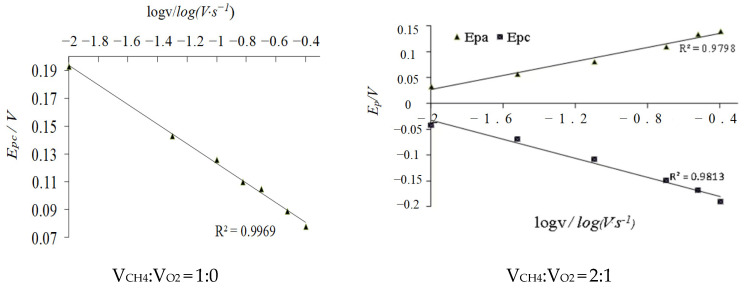
The relationship between peak potential and scanning speed (*logv*).

The CV test showed a positive correlation between the electric potential difference (Δ*E*) and scanning speeds. According to the Laviron theory, when *n*Δ*E* is higher than 200 mV, the electron transfer coefficient (*α*) can be obtained by plotting *E_p_* against *logν* [8]. Figure 3 shows the relationship between Δ*E* and *logν* in PBS solution at different volume ratios of CH_4_ and O_2_. After fitting, the linear slopes ka and kc were obtained (Figure 3); according to the formula, the former and the latter correspond to the value of 3.3 RT/(1 − *α*)*nF* and −2.3 RT/*αnF*, respectively. By the correspondence between the straight slope kc and −2.3 RT /*αnF*, the effect of the dissolved CH_4_ and O_2_ volume ratio on the electron transfer coefficient during the redox reaction on the electrode surface was obtained (Table 2).

(2) $$Epa=K+\frac{\;2.3RT}{(1-\alpha )nF}\log v$$

(3) $$Epc=K-\frac{\;2.3RT}{\alpha nF}\log v$$

#### 2.2.3. Electron Transfer Rate Constant (*k_s_*)

According to the direct electrochemical data, the peak potential difference Δ*E_p_* of the pMMO redox process was more significant than 200 mV, so the electron transfer rate constant (*k_s_*) could be calculated (Equation (4)) [9]. The dissolution results of different CH_4_ and O_2_ volume ratios on *k_s_* are shown in Table 3.

(4) $$logk_{s}\;=\alpha log(1-\alpha )+(1-\alpha )log-log(RT/nFv)-\alpha (1-\alpha )nF△E_{p}/2.3RT$$

### 2.3. Analysis of the Kinetic Behavior of pMMO Bioelectrocatalysis

This test clarified that both CH_4_ and O_2_ affect the telecom signals of CV tests. Compared with the reference, this paper’s telecom signal change ranges were better [10]. This phenomenon may be because the enzyme used in the literature was sMMO, and since sMMO had no suitable electron donor, a synthetic heppoly glycine was used as an electron transmitter for direct electrochemistry. However, the small molecule peptide Mb used in this paper is not the result of artificial synthesis; it is the natural electron transmitter of pMMO, which leads to a good range of telecom signal changes in the electrochemical test. When the electrolyte had no pMMO or Mb modified on the electrode surface, the electrical signal detected by the working electrode did not significantly change with the dissolved CH_4_ and O_2_ content.

From the results above, the kinetic behavior of pMMO bioelectrocatalysis was obtained. (1) In the CV tests at different scan speeds, the peak current showed a linear correlation with the scanning sweep (*v*, *v*^1/2^) at less than 400 mV·s^−1^, which was in line with the surface reaction dynamics model. (2) The kinetic behavior and parameter changes are shown in Table 4. The magnitude of electrical signal change was small in the detection environment containing only CH_4_ or O_2_. The pMMO electrocatalytic process was a single electron transmission process, and the electron transmission coefficient and electron transmission rate constant were relatively large. In addition, in the detection environment containing both CH_4_ and O_2_, it is speculated that the pMMO electrocatalytic CH_4_ oxidation process was a two-electron transmission process, and both the electron transmission coefficient and the electron transmission rate constant were small. Under different CH_4_ and O_2_ content test environments, these phenomena have occurred due to different catalytic viability or changes in the enzyme conformation. Moreover, the degree of pMMO binding to the Mb on the working electrode was also different.

## 3. Materials and Methods

### 3.1. Chemicals

Methanobactin (Mb) from the spent medium of Methylosinus trichosporium 3011, which was obtained from the Institute of Microbiology and Virology (Kiev, Ukraine), was grown up in a 5 L bioreactor containing 3 L copper-deficient nitrate minimal salts (NMS) medium as previously described [11]. Culture conditions for pMMO expression were carried out under the same conditions, except for adding 2 × 10^−5^ mol/L of CuSO_4_ to the medium. The isolation and quantification of Mb, preparation of the pMMO-enriched membrane fraction, and activity assay of pMMO were all performed using those previously used by the research group [12]. Deionized water was used with a conductivity of 20 μs/cm. AuCl_3_HCl·_4_H_2_O (Au% > 48%, the Shanghai No. 1 Reagent Factory, Shanghai, China) and all other chemicals were of analytical grade. Phosphate buffer solutions (0.02 M) with various pH values were prepared by mixing stock standard solutions of Na_2_HPO_4_ and NaH_2_PO_4_ and adjusting the pH with 0.1 M H_3_PO_4_ or NaOH. All solutions were made up of twice-distilled water [13].

### 3.2. Culture Preparation and Isolation and Purification of pMMO

#### 3.2.1. Fermentation Culture

Methane-oxidizing bacteria were cultured in a copper-containing medium. Methylosinus trichosporium IMV 3011 strain was cultured in a high copper inorganic salt medium (pH 7.0). The medium was dispensed in silk-spouted bottles (250 mL) with air-exchange hoses; each was 100 mL. Each bottle was sterilized in an autoclave for 30 min (121 °C, 0.1 MPa). After autoclaving, each bottle was placed on an ultra-clean bench, sterilized by ultraviolet light for 20 min, and cooled to room temperature. The inoculation volume was 10% of the culture medium (*v*/*v*), and the inoculum was pumped. When the air pressure was less than 0.09 MPa, a mixture of methane and air (*v*:*v* = 1:1 by volume) was introduced and placed in a constant-temperature shaking bed for oscillation cultivation (300 rpm, 32 °C). The mixture of gases (*v*:*v* = 1:1 by volume of methane and air) was changed every 24 h. The cultivation cycle time was 72–96 h. After the incubation, the cultures were stored in a refrigerator at 4 °C for spare parts.

#### 3.2.2. Purification of pMMO-Containing Endosomes

All the purification operations were carried out under airtight conditions at 4 °C. The fermentation broth was centrifuged at 9000 rpm for 10 min to collect the bacterial precipitate. The appropriate amount of phosphate-buffered saline (PBS) buffer solution (20 mM, pH = 7.0, containing 5 mM MgCl_2_) was added to re-solubilize. It was centrifuged again, and the operation was repeated three times to obtain the intact cells of Mb. An appropriate amount of PBS buffer solution containing (20 mM, pH = 7.0, containing 25 mM dithiothreitol) was added to the obtained cell precipitate at the ratio of 10 mL/g to make a bacterial suspension. An ultrasonic cell crusher (120 W power working for 1 s, 2 s intervals, ultrasonic crushing for 15 times) was used to crush the cells. Afterward, the cell breakage solution was centrifuged at 12,000 rpm for 30 min to remove the unbroken cells and cell debris. Then, the supernatant was ultracentrifuged at 100,000 rpm for 90 min to obtain the precipitate, which was the cytoplasmic endomembrane fraction, and was stored in liquid nitrogen at −80 °C for spare use and PBS added to re-dissolve before using.

#### 3.2.3. pMMO Specific Activity Measurement

One milliliter of pMMO inner membrane fraction (2 mg/mL) was placed in a 10 mL reaction vial, and an electron donor (hydroquinone, o-methylhydroquinone, dimethylhydroquinone, 1 mM) was added and sealed with a soft stopper. At the same time, one milliliter of air was drawn above the reaction vial with a syringe, and one milliliter of propylene was displaced into it. The reaction was conducted at 30 °C, 180 rpm, for 20 min. The amount of propylene oxide produced (retention time 1.615 min) was determined by gas chromatography (GC). The specific activity of MMO was expressed as the number of moles of propylene oxide catalyzed per minute per milligram of cell-dried weight (in nmol/(min∙mg)).

Propylene oxide was detected by using gas chromatography under the following conditions: the carrier gas was N_2_; the flow rate was 1.0 mL/min; the capillary column with a column temperature of 60 °C; the injection temperature was 180 °C; the detection temperature was 180 °C with a hydrogen flame ionization (FID) detector; and the manual injection with a sample volume of 1 μL.

### 3.3. Gold Disk Electrode Pretreatment

The gold disk electrode was immersed in the washing solution (volume ratio of concentrated H_2_SO_4_ to 30% H_2_O_2_ was 7:3) for 10~30 min, rinsed with double-distilled water, and then washed successively by ultrasonic oscillation using methanol and double-distilled water for 30 s, and then air-dried to be used.

**Figure 4:**
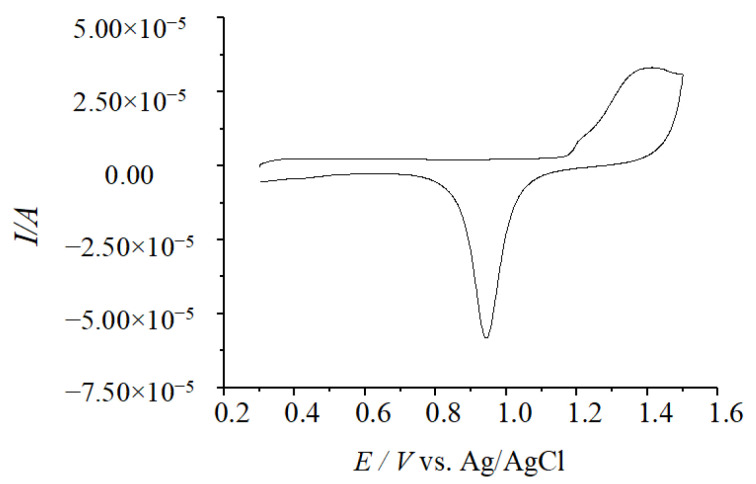
CV response of the bare Au electrode in 1 M H_2_SO_4_ solution.

(1) The gold disk electrodes were placed in 1 M H_2_SO_4_ solution, and the CV scanning test was carried out to the stable redox peak shape of gold at 100 mV·s^−1^ within the potential window from 0.0 to +1.5 V (vs. Ag/AgCl), as shown in Figure 4. Its electrochemical area was integrated from the gold oxide reduction region to obtain the reduction peak charge. The actual area of the electrode was found to be 0.06359 ± 0.0006 cm^2^ by Equation (5).

(5) A=kQ

where *Q*—integrated area, μC; k—Constant of integration, numerically 420; *A*—true area of the electrode surface where the electrochemical reaction occurs, cm^2^.

**Figure 5:**
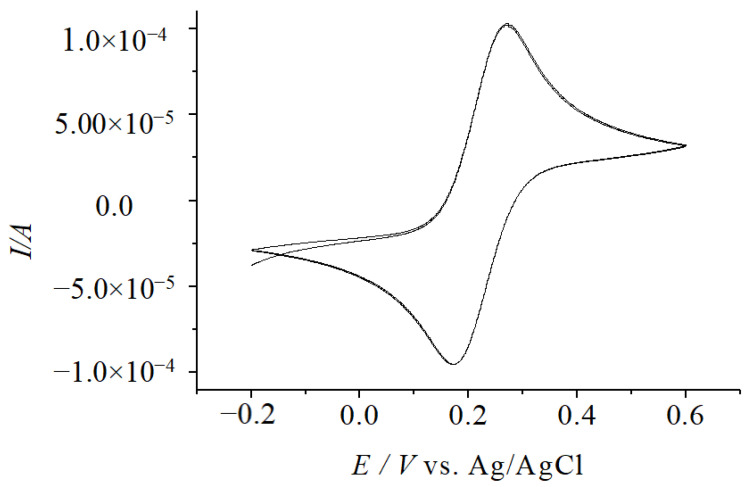
CV of the bare Au electrode in 5.00 × 10^−3^ M Fe(CN)_6_^3−/4−^ solution.

(2) At the same time, the pretreated gold disk electrodes were placed in 0.1 M KCl electrolyte containing 5.00 mM K_3_[Fe(CN)_6_] and 5.00 mM K_4_[Fe(CN)_6_]. The CV scanning was carried out within the potential window of −0.2~+0.6 V (vs. Ag/AgCl), and the sweep rate was 0.02 V·s^−1^. If the Δ*E_p_* value of the two-turn CV test pattern was below 0.1 V, it was judged to have obtained a stable redox peak shape of gold, and the preprocessing was completed, as shown in Figure 5.

### 3.4. Preparation of Pure AuNPs Sols by Chemical Method

The glassware used to prepare AuNPs was thoroughly cleaned in advance, i.e., it was first rinsed with freshly prepared aqua regia, then thoroughly rinsed with deionized water and finally dried in an oven. Pure gold gels were prepared by reducing AuCl_3_HCl·4H_2_O using sodium citrate according to the chemical method in a 250 mL round-bottomed flask with one condenser, 50 mL of l mM of AuCl_3_HCl·4H_2_O was added, and the mixture heated to boiling for about 0.5 h at a controlled voltage of less than 130 V. With vigorous stirring, 5 mL of 38.8 mM sodium citrate was added rapidly to the vortex of the solution, and the color lightened from yellowish until it changed to purplish red. It was boiled for 10 min, removed from the heat source and stirring continued for 15 min, cooled to room temperature, and refrigerated at 4 °C.

### 3.5. Preparation of AuNPs Modified with Mb (Mb-AuNPs)

The pure AuNPs solution prepared chemically was taken, and the absorption value of the AuNPs particle solution was adjusted to about 1.1 with deionized water prior to use. Mb solution was added to give a final concentration of 5 × 10^−5^ M. The mixture was then placed at 4 °C and protected from light for 12 h. The reacted solution was centrifuged at 8000 rpm for 30 min and then washed with deionized water to remove the free Mb and Au nanoparticles modified with Mb (denoted as Mb-AuNPs) were obtained [14].

### 3.6. Preparation of AuNPs with Mixed Modifications of Mb and Mercapto Compounds

Pure AuNPs solution prepared chemically was taken, and the absorption value of the AuNPs solution was adjusted with deionized water to about 1.1 before use. In total, 1 mL of this AuNPs solution was taken, to which 50 μL of Mb solution (Mb final concentration of 5 × 10^−5^ M) was added simultaneously along with specific volumes (0.5, 2.5, 5.0, 7.5, and 10.0 μL) of 0.125 mM mercaptan reagent solution (1,2-ethylenedithiol, dithiothreitol, 1,6-hexanedithiol, β-mercaptoethylamine, 2-mercaptoethanol). The solution was mixed thoroughly while dropping and stored overnight at 4 °C away from light for 12 h. The reacted solution was centrifuged (8000 rpm, 30 min) and washed with deionized water to remove the free mercaptan reagent and Mb [15] and to allow the Mb and mercaptans in solution to form compounds through coordination bonds.

### 3.7. Preparation of {Mb-AuNPs}4/Au Disk Electrodes

On the surfaces of the pretreated bare Au electrodes, 20 μL of the Mb-functionalized AuNPs solution (the Mb final concentration was 0.025 M) was added dropwise to stabilize the adsorption with the surface of the gold disk electrode by using the -SH group of the Mb-functionalized AuNPs at 4 °C for 12 h in a dark environment [16]. The electrode surfaces were rinsed with double-distilled water and dried to obtain the monolayer self-assembled modified electrodes with Mb-functionalized AuNPs. After that, these electrodes were immersed in 10 mL of a mixed solution of 75 mM EDC and 15 mM NHS to activate the -COOH group in the Mb structure on the functionalized AuNPs. Moreover, they were left to stand for 12 h in a humid and dark environment at 4 °C. After removal, the electrode surfaces were rinsed with double-distilled water and dried. At that time, 20 μL of Mb-functionalized AuNPs (Mb final concentration was 0.025 M) was added dropwise to the surfaces of the electrodes, which were left to stand for 12 h in a humid and dark environment at 4 °C. By using the −COOH group of the Mb, amide bonds were formed with the -NH_2_ group in the other Mb structure, and also reacted several times. After completing the last layer of self-assembly modification, the remaining active sites on the electrode surfaces were sealed with glycine in a closed and humid environment for 1 h (10 g/L, 4 °C) to prevent the bias caused by the nonspecific adsorption of other proteins in the test solution. The Mb-functionalized AuNPs layer-by-layer self-assembly modified electrodes ({Mb-AuNPs}n/Au) were obtained.

### 3.8. Kinetic Behavior and Analysis of pMMO Bioelectrocatalysis

Test electrodes: {Mb-AuNPs}4/Au disk modified electrodes.

Test electrolyte: A total of 10 mL of PBS (containing the pMMO inner membrane component, where the total specific activity of MMO in the electrolyte was 44.9 nmol/(min-mg)) was dissolved with different volume ratios of CH_4_ and O_2_ at 20 °C [17]. The electrolytic cell was airtight. The test electrodes were subjected to the CV test with different scanning speeds. The following four cases are among the electrolyte’s dissolved CH_4_ and O_2_ volume ratios. (1) Oxygen saturation: The O_2_ gas was passed to the PBS for 4 h, assuming the solution was saturated with O_2_ gas. The oxygen content was 9.17 mg/L (at 20 °C, at 1 standard atmosphere, 1 volume of water was saturated with 0.031 volume of dissolved O_2_). The CH_4_ and O_2_ volume ratios in the electrolyte were 0:1 (V_CH4_:V_O2_ = 0:1). (2) Methane saturation: CH_4_ gas was passed into PBS for 4 h, assuming that the solution was saturated with CH_4_ gas at this time, with the CH_4_ content of 33.5 mg/L (at 20 °C, 1 standard atmospheric pressure, 1 volume of water was saturated with 0.03 volume of CH_4_). The electrolytic solution in the CH_4_ and O_2_ volume ratio was 1:0 (V_CH4_:V_O2_ = 1:0). (3) Injecting CH_4_ gas into the PBS containing saturated oxygen with a rate of 0.1 mL/h gas for 1 h, it was assumed all incoming CH_4_ was dissolved and the same volume of O_2_ was discharged. The volume ratio of CH_4_ and O_2_ in the electrolyte was 2:1 (V_CH4_:V_O2_ = 2:1). (4) Injecting CH_4_ gas into the PBS containing saturated oxygen with a rate of 0.1 mL/h gas for 1 h, it was assumed all incoming CH_4_ was dissolved and the same volume of O_2_ was discharged. Then, the volume ratio of CH_4_ and O_2_ in the electrolyte was 1:2 (V_CH4_:V_O2_ = 1:2). The above should be noted: ① The gas was ventilated first, and then 50 μL of biologically active pMMO endosomes was injected into the electrolytic cell by using the microfeeder (the specific activity of MMO in the electrolyte was 44.9 nmol/(min-mg) in the final electrolyte); ② In consideration of the activity of pMMO, the pH of 7.0 was chosen as the test pH of the electrolyte.

Test conditions: CV tests were conducted under the above four conditions (potential window of −0.6~+0.6 V, vs. Ag/AgCl). By changing the scanning speeds, the relationship between the sweeping speeds and the redox peak current and peak potential were obtained, which led to the electrochemical kinetic related parameters during the reaction process, which were the number of transferred electrons (*n*), the electron transfer coefficient (*α*), and the electron transfer rate constant (*k_s_*), respectively.

## 4. Conclusions

The particulate methane monooxygenase (pMMO) strongly depends on the natural electron transfer path. It is prone to denaturation, which results in its redox activity centers being unable to transfer electrons with bare electrodes directly and it being difficult to observe the electrochemical response. A bionic interface with high biocompatibility and stability was created using methanobactin (Mb) as the electron transporter between gold electrodes and pMMO. The Mb-AuNPs-modified functionalized gold net electrode as a working electrode, the kinetic behaviors of pMMO bioelectrocatalysis, and the effect of Mb on pMMO were analyzed. The CV tests were performed at different scanning rates to obtain electrochemical kinetics parameters. Under a test environment only containing CH_4_ or O_2_, the value of the electron transfer coefficient (*α*) and electron transfer rate constant (*k_s_*) were more significant, relatively.

In contrast, in the test environment containing both methane and O_2_, the bioelectrocatalysis of pMMO is a two-electron transfer process with a relatively small α and *k_s_*. It follows that Mb forms a complex with pMMO. More importantly, it was experimentally demonstrated that the presence of Mb resulted in a more stable electron transfer and also played an important role in stabilizing the enzyme structure of pMMO and maintaining a specific redox state. In addition, continuous catalytic oxidation of the natural substrate methane was achieved.

## Figures and Tables

**Table 1:** Influence of the volume ratio of CH_4_ and O_2_ on the electron transfer number.

The Volume Ratio of Dissolved CH_4_ and Dissolved O_2_ (V_CH4_:V_O2_)	0:1	1:2	2:1	1:0
Actual calculated transferred electron number	0.947	1.57	1.81	1.019
Approximately considered transferred electron number	1	2	2	1

**Table 2:** Influence of the volume ratio of CH_4_ and O_2_ on the electron transfer coefficient.

The Volume Ratio of Dissolved CH_4_ and Dissolved O_2_ (V_CH4_:V_O2_)	0:1	1:2	2:1	1:0
*α*	0.7058	0.59	0.47	0.74

**Table 3:** Influence of the volume ratio of CH_4_ and O_2_ on the electron transfer rate constant.

The Volume Ratio of Dissolved CH_4_ and Dissolved O_2_ (V_CH4_:V_O2_)	0:1	1:2	2:1	1:0
*k_s_*(s^−1^)	0.5571	0.3886	0.113	0.5459

**Table 4:** Influence of CH_4_ and O_2_ volume ratio on the electron transfer rate constant.

The Volume Ratio of Dissolved CH_4_ and Dissolved O_2_ (V_CH4_:V_O2_)	The Kinetic Behavior and Parameters of pMMO Bioelectrocatalysis
0:1	The pMMO-catalyzed redox reaction was a single-electron transport process; the electron transfer coefficient was 0.7058, and the electron transfer rate constant was 0.5571 s^−1^.
1:2	The pMMO-catalyzed redox reaction was a two-electron transport process; the electron transfer coefficient was 0.59, and the electron transfer rate constant was 0.3886 s^−1^.
2:1	The pMMO-catalyzed redox reaction was a single-electron transport process; the electron transfer coefficient was 0.47, and the electron transfer rate constant was 0.113 s^−1^.
1:0	The pMMO-catalyzed redox reaction was a single-electron transport process; the electron transfer coefficient was 0.47, and the electron transfer rate constant was 0.5459 s^−1^.

## Data Availability

Data are contained within the article.
